# Cardiorespiratory fitness and effects of ubiquinol during high-altitude acclimatization and deacclimatization: The SCARF trial

**DOI:** 10.1016/j.isci.2025.112112

**Published:** 2025-02-27

**Authors:** Hailin Lv, Zhen Liu, Mengjia Sun, Shiyong Yu, Mingdong Hu, Shizhu Bian, Xiaowei Ye, Ke Wang, Hongmei Dong, Bingjie Yang, Chao Zhou, Lan Huang, Jie Yang

**Affiliations:** 1 Institute of Cardiovascular Diseases of PLA, the Second Affiliated Hospital, Army Medical University, Chongqing, China; 2 Department of Cardiology, the Second Affiliated Hospital, Army Medical University (Third Military Medical University), Chongqing, P.R. China; 3 Department of Physical Examination, the Second Affiliated Hospital, Army Medical University, Chongqing, China; 4 Department of Health Care and Geriatrics, The 941st Hospital of Joint Logistics Support Force of PLA, Sining, China; 5 953rd Hospital, Shigatse Branch, Xinqiao Hospital, Army Medical University (Third Military Medical University), Shigatse, China

**Keywords:** Kinesiology, Climatology

## Abstract

The dynamic characteristics of cardiorespiratory fitness during high-altitude acclimatization and deacclimatization are not well elucidated, and whether ubiquinol exerts beneficial effects on cardiorespiratory fitness remains debated. In this trial, 41 volunteers were randomized to receive oral ubiquinol or placebo administration, 14 days before departure to highlands. All individuals were carried to 3900 m by air and then returned to 300 m after 7 days. Cardiopulmonary exercise testing was performed at baseline, on the third day after arrival in the highlands, and on the seventh day after return. This trial revealed the dynamic characteristics of cardiorespiratory fitness during the entire high-altitude acclimatization and deacclimatization process. The short-term journey to the highlands did not significantly affect cardiorespiratory fitness or physical performance capacity after the return. Cardiovascular and respiratory recoveries were desynchronized after returning from the highlands. Ubiquinol supplementation maintained the physical performance capacity in the highlands and facilitated acclimatization to hypoxia.

Trial registration: The Chinese Clinical Trial Registry, ChiCTR2200059900, http://www.chictr.org.cn/ChiCTR2200059900.

## Highlights

• Short-term highland journey does not affect cardiorespiratory fitness after return
• Cardiovascular and respiratory recoveries are desynchronized after return
• Ubiquinol maintains physical performance capacity in the highlands
• Ubiquinol facilitates acclimatization to hypoxia

## Graphical abstract

**Figure:**
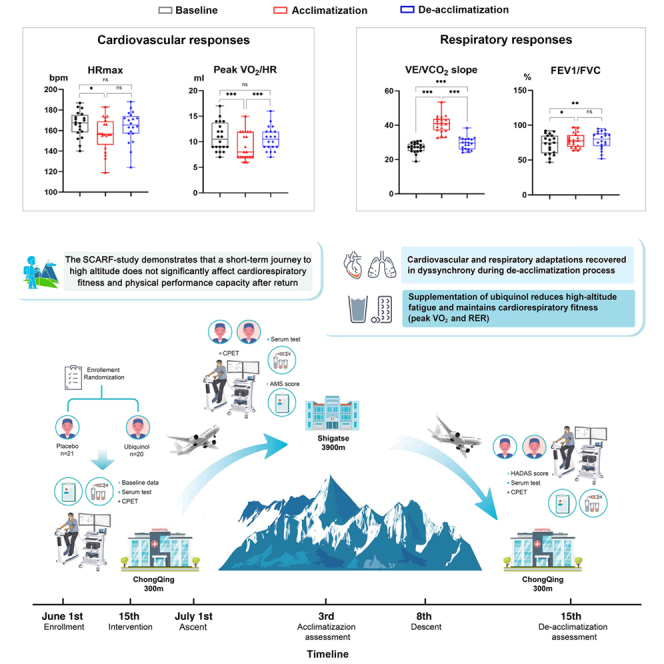

## Introduction

Hypoxia and subsequent reoxygenation are two major challenges for individuals who ascend to highlands and subsequently return to sea level within a short period. During this process, coordination of the cardiovascular and respiratory systems enables most individuals to adapt to hypoxia and reoxygenation, which also determines their acclimatization capacity under stress conditions.^1^^,^^2^ Cardiorespiratory fitness is an effective and reliable indicator for evaluating the collaborative functional capacity of the cardiovascular and respiratory systems. Hypoxia decreases cardiorespiratory fitness because of combined reduction in arterial oxygenation and increased pulmonary vascular resistance.^3^^,^^4^ Persistent chemoreceptor activation and increased sympathetic vasoconstriction activity prevents the recovery of cardiorespiratory fitness after reoxygenation.^5^ However, cardiorespiratory fitness characteristics during high-altitude acclimatization and deacclimatization are poorly understood. Clarification of these changing characteristics should benefit efforts aimed at exploring potential therapeutics to promote high-altitude acclimatization and deacclimatization.

In a recent study, we showed that optimizing myocardial energy metabolism may help prevent altitude-induced cardiorespiratory fitness impairment.^6^ Supplementation of endogenous energy metabolic intermediates or enzymes may be acceptable and widely used to maintain cardiorespiratory fitness. Ubiquinol acts as a diffusible electron carrier concentrated in tissues with a high energy turnover, such as the myocardium and skeletal muscle.^7^ In patients with myocardial ischemia and hypoxia, ubiquinol supplementation increases aerobic metabolic efficiency and raises the anaerobic threshold, reducing the need for anaerobic energy production at the same workload.^8^^,^^9^ Therefore, we hypothesized that (1) a short-term high-altitude exposure does not significantly affect the cardiorespiratory fitness and physical performance capacity after return; (2) cardiovascular and respiratory adaptations recover in dyssynchrony during the deacclimatization process; and (3) ubiquinol supplementation reduces high-altitude fatigue and maintains cardiorespiratory fitness.

In this prospective clinical trial, we aimed to elucidate cardiorespiratory fitness characteristics, as well as cardiovascular and respiratory responses during high-altitude acclimatization and deacclimatization. Based on follow-up evaluations conducted at different altitudes, we explored the effects of ubiquinol supplementation on cardiorespiratory fitness and high-altitude acclimatization- and deacclimatization-associated symptoms.

## Results

### Study population and characteristics

Forty-one eligible volunteers participated in the study from June 1 to June 15, 2022. Two patients did not complete the trial: one from the placebo group due to thoracic discomfort and an abnormal electrocardiograph during cardiopulmonary exercise testing (CPET) at highland, and one from the ubiquinol group who withdrew early. Consequently, efficacy analyses were based on data from 39 participants (20 placebo and 19 ubiquinol) who completed the CPET. At baseline, hardly any differences between the two groups in age, sex distribution, body mass index, incidence of acute mountain sickness (AMS), and high-altitude deacclimatization syndrome (HADAS) score existed in the final analysis (Table 1). After intervention, the serum ubiquinol concentration was significantly higher in the ubiquinol group than in the placebo group (201.4 ± 49.2 vs. 102.8 ± 45.8 nmol/L, *p* < 0.001), indicating good compliance of volunteers in the ubiquinol group (Table 1 and Figure S1).

**Table 1:** Baseline characteristics and symptom scores of the participants

Variables	Total (*N* = 39)	Placebo (*n* = 20)	Ubiquinol (*n* = 19)	P
Age, years	31.6 ± 6.5	32.3 ± 6.2	31.0 ± 6.9	0.522
Male	14 (35.9)	8 (40.0)	6 (31.6)	0.589
BMI, kg/m^2^	21.8 ± 2.8	21.9 ± 2.3	21.7 ± 3.3	0.694
Alcohol	9 (23.1)	7 (35.0)	2 (10.5)	0.073
Tobacco	5 (12.8)	1 (5.0)	4 (21.1)	0.139
AMS^a^ (%)	29 (74.4)	15 (75)	14 (73.7)	0.926
LLS score^a^	3.4 ± 2.0	3.5 ± 2.3	3.3 ± 1.6	0.805
HADAS score^b^	6.9 ± 2.6	7.5 ± 2.8	6.3 ± 2.4	0.182
Ubiquinol^a^ (nmol/L)	150.8 ± 68.5	102.8 ± 45.8	201.4 ± 49.2	<0.001

Values are means ± standard deviation or number (%). The Chi-square test, Mann-Whitney *U*-test, and independent-samples *t* test (two-sided) were used to statistically compare continuous and categorical variables. AMS, acute mountain sickness; BMI, body mass index; HADAS, high-altitude de-acclimatization syndrome; LLS, Lake Louise scoring.

a Indicates measurements during high-altitude acclimatization.

b Indicates measurements during high-altitude deacclimatization.

### Cardiorespiratory fitness

During CPET in the lowlands, after arrival in the highlands, and after return to the lowlands, all participants attained maximal volitional effort (mean respiratory exchange ratio (RER) reaching 1.10 in three measurements, Table 2). The VO_2_max (25.8 ± 5.9 vs. 30.5 ± 5.1 mL/min/kg, *p* < 0.001) and peak metabolic equivalents (METs, 7.9 ± 1.7 vs. 9.3 ± 1.4, *p* < 0.001) decreased during high-altitude acclimatization. In addition to maximal exercise capacity, the submaximal parameters also decreased (VO_2_ at AT, VO_2_ at the respiratory compensation point [RCP], and VO_2_/work rate [WR] slope; Table 2). These parameters recovered to baseline within 7 days after returning to the lowlands (VO_2_max: 29.8 ± 5.9 vs. 30.5 ± 5.1 mL/min/kg, *p* = 0.152; peak METs: 9.1 ± 1.6 vs. 9.3 ± 1.4, *p* = 0.081; VO_2_ at AT: 15 [14–17] vs. 16 [13–17], *p* = 0.963; VO_2_ at RCP: 26.1 ± 5.1 vs. 27.4 ± 4.7, *p* = 0.031; VO_2_/WR slope: 9.2 ± 1.1 vs. 9.4 ± 1.0, *p* = 0.177). Peripheral blood oxygen saturation (SpO_2_) decreased significantly at rest and exercise during acclimatization and recovered to baseline levels after returning to the lowlands (Table 2).

**Table 2:** Cardiorespiratory fitness during high-altitude acclimatization and deacclimatization

Variables	Baseline (*n* = 39)	Acclimatization (*n* = 39)	De-acclimatization (*n* = 39)	P1	P2	P3
Peak VO_2_ (mL/min/kg)	30.5 ± 5.1	25.8 ± 5.9	29.8 ± 5.9	<0.001	0.152	<0.001
Peak METS	9.3 ± 1.4	7.9 ± 1.7	9.1 ± 1.6	<0.001	0.081	<0.001
Peak RER	1.15 ± 0.10	1.10 ± 0.09	1.10 ± 0.06	<0.001	<0.001	0.955
RER at AT	0.84 (0.81–0.87)	0.88 (0.86–0.91)	0.85 (0.81–0.88)	<0.001	0.290	<0.001
VO_2_ at AT (mL/min/kg)	16.0 (13.0–17.0)	14.0 (13.0–16.0)	15.0 (14.0–17.0)	0.024	0.963	0.022
VO_2_ at RCP (mL/min/kg)	27.4 ± 4.7	22. 7 ± 5.4	26.1 ± 5.1	<0.001	0.031	<0.001
VO_2_/WR slope (mL/min/W)	9.4 ± 1.0	8.2 ± 1.2	9.2 ± 1.1	<0.001	0.177	<0.001
SpO2 at rest (%)	97.0 (96.0–98.0)	83.0 (81.0–86.0)	97.0 (96.0–97.0)	<0.001	0.462	<0.001
SpO2 at AT (%)	96.0 (95.0–97.0)	82.0 (77.0–84.0)	97.0 (96.0–98.0)	<0.001	0.147	<0.001
SpO2 at RCP (%)	96.0 (95.0–98.0)	80.0 (78.0–83.0)	97.0 (94.0–97.0)	<0.001	0.899	<0.001
Peak SpO2 (%)	95.0 (94.0–96.0)	80.0 (78.0–85.0)	95.0 (91.0–97.0)	<0.001	0.716	<0.001

Values are means ± standard deviation or median (interquartile range).

P1, difference between baseline and acclimatization; P2, difference between baseline and deacclimatization; P3, difference between acclimatization and deacclimatization. VO_2_, oxygen uptake; METs, metabolic equivalents; RER, respiratory exchange ratio; AT, anaerobic threshold; RCP, respiratory compensation point; WR, work rate; SpO2, peripheral blood oxygen saturation.

As shown in Table 3, no significant differences were observed between the placebo and ubiquinol groups in baseline cardiorespiratory fitness variables. However, during high-altitude acclimatization, the VO_2_max (27.5 ± 5.1 vs. 24.3 ± 6.3 mL/min/kg, *p* = 0.040) and peak METs (8.4 ± 1.5 vs. 7.3 ± 1.8, *p* = 0.035) were notably higher in the ubiquinol group than in the placebo group. Peak RER decreased in the placebo group, whereas it was preserved in the ubiquinol group (1.16 ± 0.06 vs. 1.12 ± 0.06, *p* = 0.031). Although SpO_2_ at RCP decreased in the ubiquinol group during deacclimatization (96.0 [94.0–97.0] vs. 97.0 [96.0–98.0], *p* = 0.033), such unfavorable effects of ubiquinol on other cardiorespiratory fitness variables were not observed during the deacclimatization period.

**Table 3:** Effects of ubiquinol during high-altitude acclimatization and deacclimatization

Variables	Baseline			Acclimatization			De-acclimatization			P1	P2	P3	interaction effect
	Placebo (*n* = 20)	Ubiquinol (*n* = 19)	P	Placebo (*n* = 20)	Ubiquinol (*n* = 19)	P	Placebo (*n* = 20)	Ubiquinol (*n* = 19)	P				
VO_2_max (mL/min/kg), mean (SD)	30.2 ± 5.4	30.9 ± 4.9	0.460	24.3 ± 6.3	27.5 ± 5.1	0.040	29.5 ± 5.8	30.2 ± 6.1	0.494	<0.001	0.152	<0.001	0.037
Peak METs, mean (SD)	9.2 ± 1.5	9.5 ± 1.4	0.449	7.3 ± 1.8	8.4 ± 1.5	0.035	8.9 ± 1.6	9.2 ± 1.7	0.451	<0.001	0.081	<0.001	0.036
Peak RER, mean (SD)	1.21 ± 0.07	1.21 ± 0.09	0.843	1.12 ± 0.06	1.16 ± 0.06	0.031	1.19 ± 0.07	1.22 ± 0.08	0.271	<0.001	0.831	<0.001	0.419
RER at AT, median (IQR)	0.84 (0.81–0.87)	0.83 (0.78–0.88)	0.453	0.89 (0.86–0.92)	0.88 (0.86–0.91)	0.422	0.85 (0.81–0.88)	0.85 (0.80–0.88)	0.869	<0.001	0.290	<0.001	0.866
VO_2_ at AT (mL/min/kg), median (IQR)	15.0 (12.0–17.0)	16.0 (15.0–17.0)	0.850	13.5 (12.0–16.8)	15.0 (13.0–16.0)	0.351	16.0 (13.3–17.0)	15.0 (14.0–18.0)	0.958	0.024	0.963	0.022	0.683
VO_2_ at RCP (mL/min/kg), mean (SD)	26.5 ± 4.2	28.3 ± 5.1	0.189	21.6 ± 5.4	23.8 ± 5.3	0.127	25.0 ± 5.1	27.4 ± 4.9	0.091	<0.001	0.031	<0.001	0.0836
VO_2_/WR slope (mL/min/W), mean (SD)	9.2 ± 0.9	9.6 ± 1.0	0.129	8.2 ± 1.4	8.3 ± 1.1	0.558	8.8 ± 1.1	9.5 ± 0.9	0.025	<0.001	0.177	<0.001	0.229
SpO2 at rest (%)	97.0 (95.3–98.0)	97.0 (96.0–98.0)	0.943	83.5 (81.3–86.5)	82.0 (79.0–86.0)	0.225	97.0 (96.0–97.8)	97.0 (96.0–97.0)	0.496	<0.001	0.462	<0.001	0.200
SpO2 at AT (%)	96.0 (95.0–97.0)	97.0 (96.0–98.0)	0.288	83.0 (78.3–84.0)	80.0 (77.0–84.0)	0.489	97.0 (96.0–97.8)	97.0 (96.0–98.0)	0.945	<0.001	0.147	<0.001	0.069
SpO2 at RCP (%)	96.0 (95.0–97.8)	96.0 (94.0–98.0)	0.977	81.5 (79.0–84.0)	80.0 (76.0–83.0)	0.259	97.0 (96.0–98.0)	96.0 (94.0–97.0)	0.033	<0.001	0.899	<0.001	0.810
Peak SpO2 (%)	95.0 (94.3–96.0)	96.0 (93.0–97.0)	0.251	81.0 (79.0–85.0)	80.0 (77.0–85.0)	0.437	96.0 (95.0–97.0)	95.0 (90.0–97.0)	0.168	<0.001	0.716	<0.001	0.654

Values are means ± SD or median (IQR). Repeated-measures analysis of variance was used if the data were normally distributed; otherwise, generalized estimating equations were used.

P1, mean difference between baseline and acclimatization; P2, mean difference between baseline and deacclimatization; P3, mean difference between acclimatization and deacclimatization; AT, anaerobic threshold; METs, metabolic equivalents; RCP, respiratory compensation point; RER, respiratory exchange ratio; VO_2_, oxygen uptake; WR, work rate; SD, standard deviation; IQR, interquartile range; SpO_2_, peripheral blood oxygen saturation.

The data separated by sex are presented in Table S1. The VO_2_max in females was lower than that in males at baseline (28.9 ± 4.3 vs. 33.4 ± 5.3, *p* = 0.006) and during acclimatization (24.1 ± 5.4 vs. 28.9 ± 5.8, *p* = 0.013) and deacclimatization (28.0 ± 4.8 vs. 33.1 ± 6.3, *p* = 0.007). Some other parameters in females decreased during acclimatization as well, included VO_2_ at RCP (21.3 ± 4.8 vs. 25.1 ± 5.7, *p* = 0.030), VO_2_/WR slope (7.7 ± 1.1 vs. 9.1 ± 0.9, *p* < 0.001), and SpO_2_ at rest (81.0 [79.5–84.0] vs. 85.5 [83.8–89.0], *p* = 0.002). However, SpO_2_ at AT in females was higher than that in males during deacclimatization (97.0 [96.5–98.0] vs. 96.0[95.0–97.0], *p* = 0.010, Table S1).

### Cardiovascular responses

**Figure 1:**
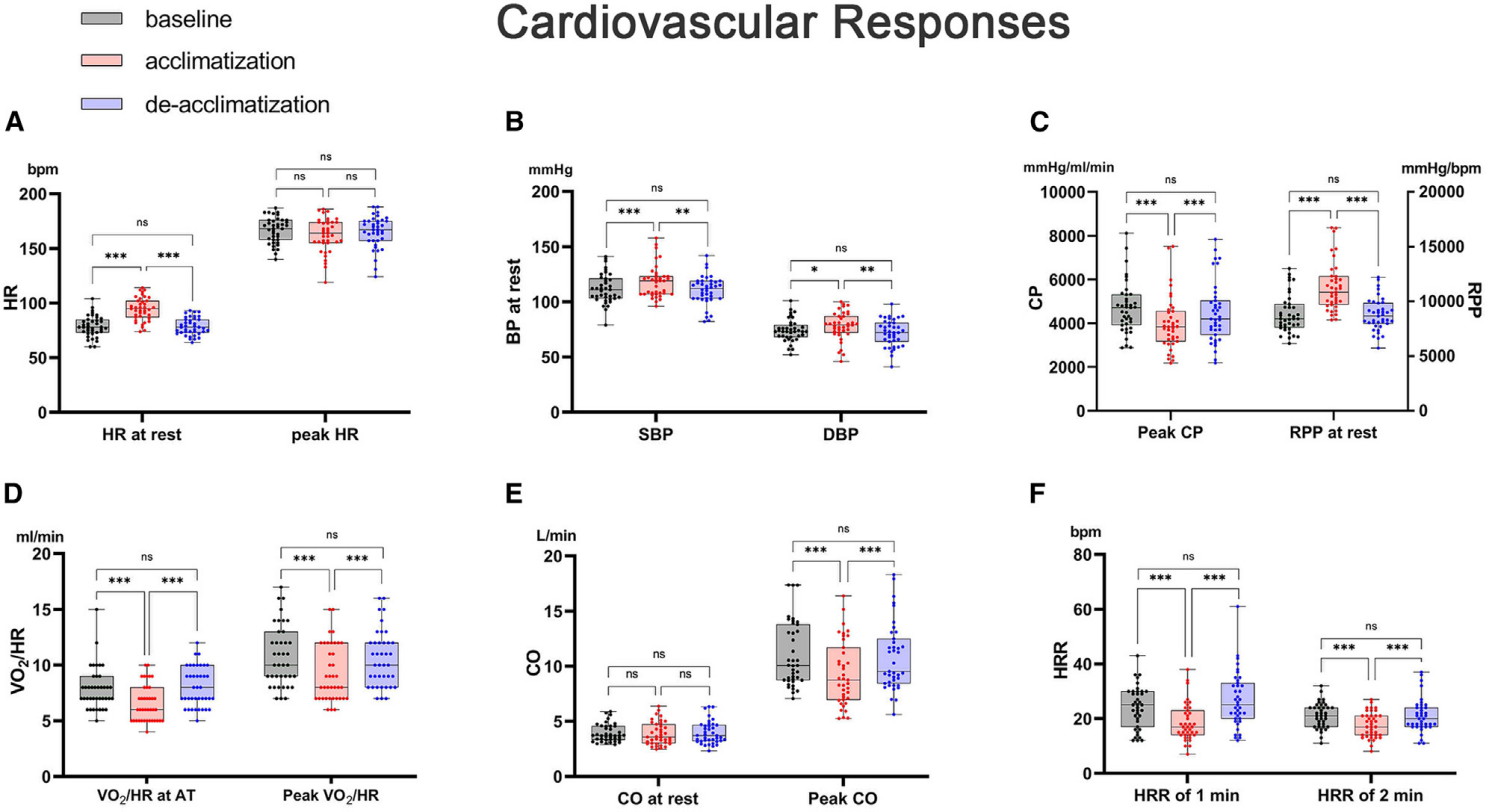
Cardiovascular response during acclimatization and deacclimatization (A–F) The index of cardiovascular responses changed during acclimatization and deacclimatization, including HR (A), BP (B), CP and RPP (C), VO_2_/HR (D), CO (E), and HRR (F). Data are represented as means ± standard deviation; Repeated-measures analysis of variance was used if the data were normally distributed; ns, *p* > 0.05; ∗*p* < 0.05; ∗∗*p* < 0.01; ∗∗∗*p* < 0.001; *n* = 39; otherwise, generalized estimating equations were used. AT, anaerobic threshold; BP, blood pressure; CO, cardiac output; CP, circulatory power; HR, heart rate; HRR, heart rate reserve; VO_2_, oxygen uptake.

As shown in Figure 1 and Table S2, in the resting condition at highlands, the values of heart rate (HR, Figure 1A), systolic blood pressure (SBP, Figure 1B), diastolic blood pressure (DBP, Figure 1B), and rate pressure product (RPP, Figure 1C) were increased. All these parameters recovered to baseline levels after return to lowlands (HR: 78.8 ± 9.6 vs. 78.6 ± 7.8 bpm, *p* = 0.870; SBP: 111.9 ± 13.4 vs. 110.3 ± 13.2 mmHg, *p* = 0.506; DBP: 74.1 ± 10.2 vs. 71.8 ± 12 mmHg, *p* = 0.233; RPP: 8853 ± 1751 vs. 8780 ± 1462 mmHg·bpm, *p* = 0.112). However, cardiac output (CO, Figure 1D) at rest was almost unchanged during the acclimatization and deacclimatization processes.

For maximal and submaximal exercise, peak circulatory power (CP, Figure 1C), peak CO (Figure 1D), oxygen pulse (VO_2_/HR) at AT (Figure 1E), peak oxygen pulse (Figure 1E), and HR recovery (HRR) at 1 and 2 min (Figure 1F) decreased during high-altitude acclimatization and were restored during deacclimatization (peak CP: 4.7 ± 1.2 vs. 4.5 ± 1.4 mmHg L/min, *p* = 0.107; peak oxygen pulse: 10.8 ± 2.7 vs. 10.6 ± 2.5 mL, *p* = 0.167; peak CO: 11.0 ± 2.9 vs. 10.8 ± 3.1 L/min, *p* = 0.180; HRR at 1 min: 24.1 ± 7.7 vs. 26.8 ± 10.1 bpm, *p* = 0.091; HRR at 2 min: 21.2 ± 4.6 vs. 21.2 ± 6.1 bpm, *p* = 0.936, Table S2). Notably, the peak HR value in the ubiquinol group was largely maintained in the highlands compared with that in the placebo group (168.16 ± 12.33 vs. 156.25 ± 15.93, *p* = 0.013), although the peak HR value in the two groups increased to the baseline levels upon return (Table S2).

These data indicate that the cardiovascular responses during acclimatization recovered to baseline levels after returning to the lowlands.

### Respiratory responses

**Figure 2:**
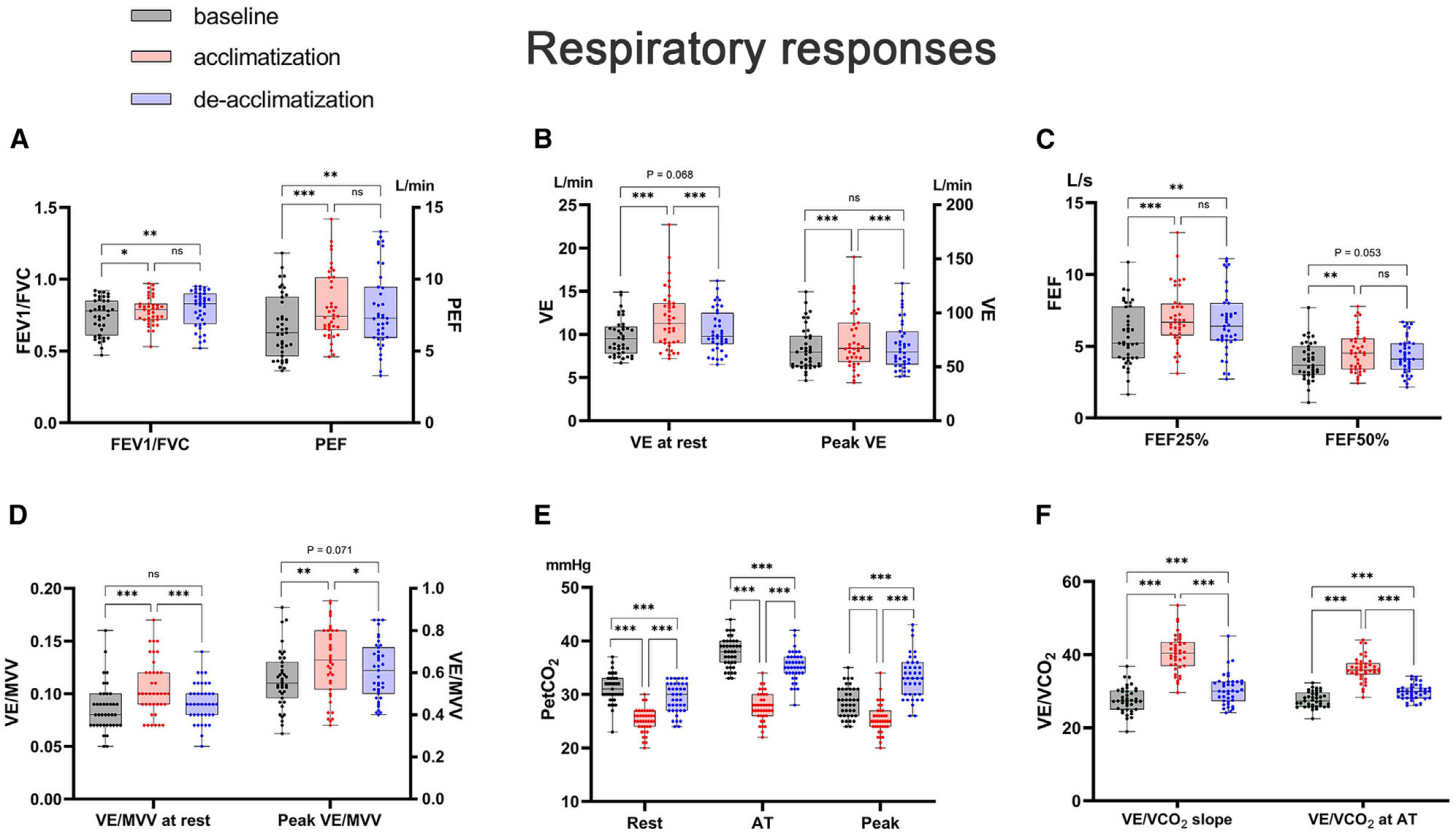
Respiratory response during acclimatization and deacclimatization (A–F) The index of respiratory responses changed during acclimatization and deacclimatization, including FEV_1_/FVC and PEF (A), VE (B), FEF (C), VE/MVV (D), PetCO_2_ (E), and VE/VCO_2_ (F). Data are represented as means ± standard deviation; Repeated-measures analysis of variance was used if the data were normally distributed; ns, *p* > 0.05; ∗*p* < 0.05; ∗∗*p* < 0.01; ∗∗∗*p* < 0.001; *n* = 39; otherwise, generalized estimating equations were used. AT, anaerobic threshold; FEF, forced expiratory flow; FEV1, forced expiratory volume in 1 s; FVC, forced vital capacity; HR, heart rate; MVV, maximum voluntary ventilation; PetCO2, end-tidal partial pressure of carbon dioxide; PEF, peak expiratory flow; RPP, rate pressure product; VE, minute ventilation; VCO2, carbon dioxide uptake.

Acute high-altitude exposure caused tachypnea, resulting in increased forced expiratory volume in 1 s/forced vital capacity (FEV_1_/FVC, Figure 2A), peak expiratory flow (PEF, Figure 2A), minute ventilation (VE, Figure 2B), forced expiratory flow (FEF 25% and 50%; Figure 2C), VE/maximum voluntary ventilation (VE/MVV, Figure 2D), MVV (Figure 2D), and end-tidal partial pressure of carbon dioxide (PetCO_2_, Figure 2E and Table S3). Moreover, acute hypoxia impaired gas exchange efficiency, as indicated by the increased VE/carbon dioxide uptake (VCO_2_) slope (Figure 2F), VE/oxygen uptake (VO_2_), and VE/VCO_2_ (Table S3).

In contrast to the cardiovascular responses, the respiratory responses did not recover to the baseline after return to the lowlands. Ventilation function was maintained after return, represented by FEV_1_/FVC (74.3% ± 12.6% vs. 79.6% ± 12.4%, *p* = 0.006, Figure 2A and Table S3), PEF (6.7 ± 2.2 vs. 7.9 ± 2.7 L/s, *p* = 0.001, Figure 2A and Table S3), FEF 25% (5.7 ± 2.0 vs. 6.7 ± 2.2 L/s, *p* = 0.002, Figure 2C and Table S3), and PetCO_2_ (31.1 ± 2.7 vs. 29.4 ± 2.8 mmHg, *p* = 0.006, Figure 2E and Table S3), whereas the gas exchange function persisted (indicated by VE/VCO_2_ slope: 27.7 ± 3.5 vs. 30.5 ± 4.2, *p* < 0.001; VE/VCO_2_ at AT: 27.7 ± 2.1 vs. 29.8 ± 2.1, *p* < 0.001, Figure 2F and Table S3) and differed significantly after return to the lowlands.

### Acute mountain sickness and high-altitude deacclimatization syndrome

During high-altitude acclimatization, 15 of 20 participants in the placebo group and 14 of 19 participants in the ubiquinol group experienced AMS (Table 1), with the highest AMS scores recorded on the first day of arrival, followed by a subsequent decrease over subsequent days (Figures S2A and S2B). The incidence of AMS-related symptoms, including headache, gastrointestinal distress, fatigue/weakness, and dizziness/vertigo, in the ubiquinol group was similar to that in the placebo group over the following 7 days (Figures S2C–S2F). However, fatigue score was lower in the ubiquinol group (Figure S2E). Fatigue scores were negatively correlated with serum ubiquinol concentrations (Figure S2G) on day 3 after arrival. Additionally, compared with the placebo, ubiquinol treatment had little effect on the HADAS score.

### Changes in circulating markers and correlations

**Figure 3:**
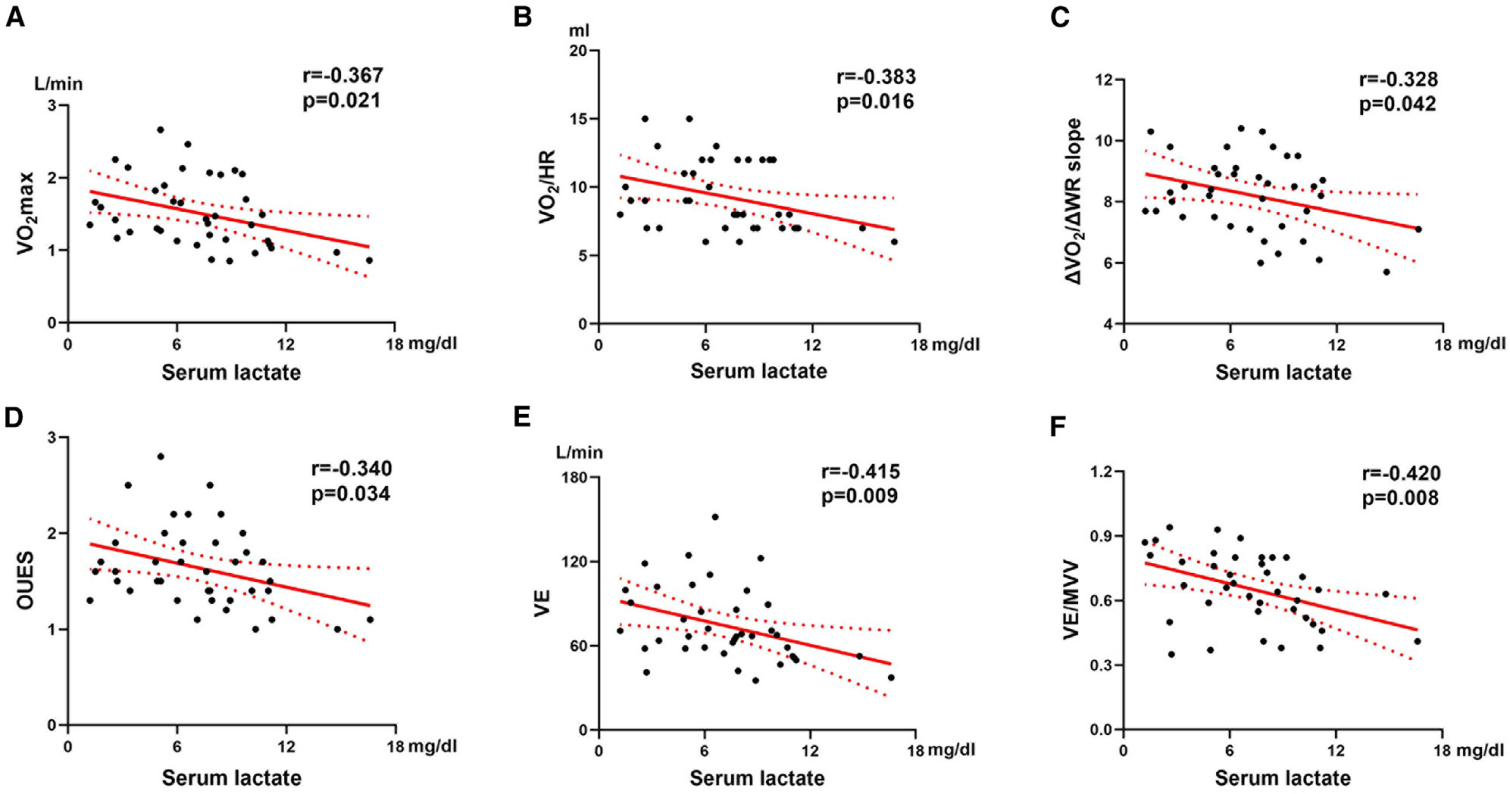
Serum lactate concentration in the highlands is negatively correlated with cardiorespiratory fitness; *n* = 39 (A–F) There were correlations between serum lactate levels and VO_2_max (A), VO_2_/HR (B), ΔVO_2_/ΔWR (C), OUES (D), VE (E), and VE/MVV (F). Correlation analyses between continuous variables were performed using Pearson’s correlation coefficient or Spearman’s rank. VO_2_max, maximum oxygen uptake; VO_2_/HR, oxygen pulse; VO_2_/WR, oxygen uptake/work rate; OUES, oxygen uptake efficiency slope; VE, minute ventilation; VE/MVV, minute ventilation/maximum voluntary ventilation.

To investigate the clinical relevance of autonomic nervous system function and circulating metabolic substances in the context of cardiorespiratory fitness, blood samples were collected at rest during the three visits. Hypoxia activated the sympathetic nervous system, as reflected by elevated norepinephrine and epinephrine levels (Table 4). Additionally, the renin-angiotensin-aldosterone system was activated, as indicated by increased plasma renin activity, angiotensin II, and angiotensin-converting enzyme 2 (Table 4). However, these increases were not restored after returning to the lowlands. Moreover, the levels of metabolic substrates and metabolites, including lactate, nonesterified fatty acids, and low-density lipoprotein-cholesterol, decreased after exposure to hypoxia; however, only lactate levels were restored to baseline after returning to the lowlands (Table 4). Notably, the serum lactate concentration during rest in the highlands correlated negatively with VO_2_max (*r* = −0.367, *p* = 0.021, Figure 3A), VO_2_/HR (*r* = −0.383, *p* = 0.016, Figure 3B), VO_2_/WR slope (*r* = −0.328, *p* = 0.042, Figure 3C), oxygen uptake efficiency slope (*r* = −0.340, *p* = 0.034, Figure 3D), VE (*r* = −0.415, *p* = 0.009, Figure 3E), and VE/MVV (*r* = −0.420, *p* = 0.008, Figure 3F) on CPET.

**Table 4:** Blood biochemical parameters during high-altitude acclimatization and deacclimatization in placebo and ubiquinol groups

Variables	Baseline			Acclimatization			De-acclimatization			P1	P2	P3	interaction effect
	Placebo (*n* = 20)	Ubiquinol (*n* = 19)	P	Placebo (*n* = 20)	Ubiquinol (*n* = 19)	P	Placebo (*n* = 20)	Ubiquinol (*n* = 19)	P				
Norepinephrine (ng/mL), mean (SD)	242.7 ± 59.4	261.1 ± 64.5	0.359	267.3 ± 179.1	312.5 ± 161.9	0.414	318.9 ± 150.7	436.9 ± 145.9	0.018	0.179	<0.001	0.023	0.173
Epinephrine (ng/mL), mean (SD)	808 ± 279	793 ± 277	0.862	1249 ± 675	1213 ± 740	0.873	1320 ± 575	1292 ± 560	0.878	0.001	<0.001	0.648	0.994
PRA (ng/mL), median (IQR)	43.5 (27.2–58.4)	40.4 (33.2–58.5)	0.777	54.2 (40.5–74.9)	51.2 (24–65.4)	0.241	49.9 (22–76.5)	35.7 (20.7–70.9)	0.666	0.021	0.527	0.200	0.505
Angiotensin II (ng/mL), median (IQR)	95.9 (81.1–123.0)	110.7 (92.2–118.2)	0.835	130.7 (89.0–173.6)	137.3 (70.9–184.9)	0.972	120.8 (79.6–193.8)	91.1 (58.2–121.2)	0.095	0.002	0.101	0.339	0.313
ACE2 (ng/mL), mean (SD)	7.7 ± 2.3	8.0 ± 2.1	0.754	8.8 ± 2.6	9.9 ± 3.7	0.269	9.1 ± 3.5	7.9 ± 3.0	0.259	0.021	0.336	0.238	0.213
Lactate (mg/dL), median (IQR)	13.2 (11.9–17.5)	13.9 (11.4–17)	0.865	8.6 (6.1–10.9)	6.0 (3.3–7.8)	0.003	14.4 (11.1–18.2)	10.6 (7.8–16)	0.015	<0.001	0.095	<0.001	0.027
NEFA (mmol/L), median (IQR)	2.1 (1.8–2.8)	2.1 (1.8–2.8)	0.434	2.0 (1.7–2.9)	1.8 (1.4–2.0)	0.001	1.6 (1.5–1.8)	1.4 (1.3–1.5)	<0.001	0.004	<0.001	<0.001	0.113
LDL-C (mmol/L), mean (SD)	2.9 ± 0.8	2.5 ± 0.8	0.126	2.6 ± 0.9	2.3 ± 0.7	0.358	2.2 ± 0.6	2.2 ± 0.6	0.793	0.027	<0.001	0.013	0.234

Values are means ± SD or median (IQR). Repeated-measures analysis of variance was used if the data were normally distributed; otherwise, generalized estimating equations were used.

P1, mean difference between baseline and acclimatization; P2, mean difference between baseline and deacclimatization; P3, mean difference between acclimatization and deacclimatization; ACE, angiotensin-converting enzyme; LDL-C, low-density lipoprotein-cholesterol; NEFA, nonesterified fatty acid; PRA, plasma renin activity; SD, standard deviation; IQR, interquartile range.

## Discussion

This clinical trial demonstrated changes in cardiorespiratory fitness during high-altitude acclimatization and deacclimatization. Short-term journeys to the highlands did not significantly affect cardiorespiratory fitness or physical performance capacity after the return. In addition, cardiovascular and respiratory recoveries were desynchronized during deacclimatization. Notably, ubiquinol supplementation maintained the physical performance capacity in the highlands and facilitated acclimatization to hypoxia.

### Cardiorespiratory acclimatization

Cardiorespiratory fitness reflects the integrated capacity of the cardiovascular and respiratory systems to transport oxygen from the atmosphere to muscle mitochondria during physical exercise.^10^^,^^11^^,^^12^^,^^13^ In the highlands, hypobaric hypoxia-induced cardiorespiratory fitness reduction poses a marked challenge, particularly for individuals engaging in high-intensity physical activity.^14^ As the partial pressure of atmospheric oxygen decreases, SpO_2_, which is sensed by the peripheral chemoreceptors, decreases. The peripherally mediated hypoxic ventilation response (HVR) is activated in response to hypoxemia, resulting in increased ventilation and partial restoration of oxygenation. However, hyperventilation reduces the partial pressure of carbon dioxide, leading to respiratory alkalosis, which inhibits both central and peripheral chemoreceptors and suppresses the HVR.^15^ Moreover, previous studies have found that the HVR is associated with SpO_2_, while SpO_2_ is associated with VO_2_max at high altitudes.^16^ Therefore, we surmised that effective HVR might be beneficial for improving the exercise capacity.

The reference values of CPET variables are affected by several factors, including age, sex, ethnicity, body surface area, environment, lifestyle habits, and exercise style. Some studies have shown that the East Asian populations have a significantly lower VO_2_max than the European and American populations.^17^^,^^18^^,^^19^^,^^20^ Moreover, our results are consistent with those of a previous study in which the CPET characteristics were assessed for 17,802 young Chinese subjects and a VO_2_max reference value of 28.92 ± 5.97 mL/kg/min was proposed.^21^ Therefore, the lower level of VO_2_max may be attributed to the inclusion of East Asian population, high proportion of females, and low body surface area. Furthermore, previous studies have indicated that cardiorespiratory fitness (evaluated by VO_2_max) linearly decreases by approximately 7% for every 1,000 m of an additional 700-meter elevation above sea level and descends in an accelerated curve over 6,300 m.^22^^,^^23^ Our clinical trial demonstrated that cardiorespiratory fitness decreased approximately by 21% (evaluated by VO_2_max) in the placebo group and by nearly 11% in the ubiquinol group after acute ascent to an altitude of 3,900 m.

Our recent research suggested that physiological responses after submaximal exercise and genetics could predict high-altitude cardiorespiratory fitness impairment (evaluated by physical work capacity 170 [PWC170]).^24^ However, the estimation of cardiorespiratory fitness by the PWC170 was less accurate than that by the CPET, which has been widely used to evaluate and guide physical exercise during cardiac rehabilitation.^10^ In the present study, the CPET data showed that VO_2_ at AT decreased in the highlands, but the RER at AT increased compared to those in the lowlands, indicating that a lower exercise intensity would result in anaerobic metabolism and fatigue in the highlands. Therefore, in the highlands, cardiac rehabilitation exercises require less intensity and a narrower exercise window for AT and RCP.

### Cardiorespiratory deacclimatization

Cardiorespiratory fitness during deacclimatization has not been thoroughly studied in individuals returning to the lowlands after short-term high-altitude exposure. Previous studies have shown that after returning to the lowlands, most individuals with prolonged high-altitude exposure experience HADAS and show oxygen saturation lower than the baseline levels, together with abnormal myocardial enzyme levels.^25^ These levels are gradually restored to the baseline levels in several weeks. However, the cardiorespiratory fitness during the process of deacclimatization following short-term high-altitude exposure remains unclear. We found that the cardiorespiratory fitness (evaluated using VO_2_max, VO_2_ at AT, VO_2_/WR slope, SpO_2_, and peak METs) returned to baseline levels after short-term high-altitude exposure. This indicated that hypobaric hypoxia-induced impairment of cardiorespiratory fitness could recover quickly after returning to the lowlands. Additionally, CO and CP were restored after return. This indicates that short-term exposure to high altitudes results in reversible impairment of the cardiac pumping function.

We believe that a high metabolic state after returning to the lowlands was responsible for maintaining the cardiorespiratory fitness during the deacclimatization process, which was manifested by the increase in insulin, adiponectin, and leptin levels. In addition, both the ventilation function, represented by FEV1/FVC, PEF, and FEF, and gas exchange function represented by the VE/VCO_2_ slope persisted after return; the persisting respiratory responses after return may as well account for the recovered cardiorespiratory fitness. Moreover, hypoxia promotes the secretion of erythropoietin and stimulates bone marrow hematopoiesis. The increase in the number of circulating red blood cells and hemoglobin levels enhanced the oxygen transport capacity, which might have accounted for the recovered cardiorespiratory fitness during the deacclimatization process. Therefore, a short-term journey to a highland did not significantly affect the cardiorespiratory fitness or physical performance capacity after the return.

The peculiarities of cardiorespiratory coordination largely determine individual reserves and physical capacity. In our study, the respiratory responses, unlike the cardiovascular responses, persisted after the return to the lowlands. This may be due to persistent chemoreceptor activation, which, in turn, activates the sympathetic nervous system during deacclimatization. Repeated episodes of transient hypoxia can induce a distributed “memory” in the brainstem and spinal cord circuits, expressed as persistently increased respiratory drive.^26^^,^^27^ In this study, the hypobaric-hypoxia adaptation induced a similar “memory” and enhanced the sensibility of the carotid body for a long period, which promoted hyperventilation and persistently decreased PetCO_2_ after return to the lowlands. Although these physiological adaptations may account for the desynchronized recovery of the cardiovascular and respiratory systems during deacclimatization, the specific mechanism remains unclear.

### Serum lactate

After at least one week of acclimatization to high altitudes, serum lactate levels remain lower than those at the sea level, a phenomenon known as the lactate paradox.^28^ Changes in serum lactate concentrations within the first week of acute high-altitude exposure remain controversial. In some studies, lactate levels increased on the first day of high-altitude exposure, whereas in others the levels were maintained.^29^ We believe that the differences in these data might be related to the rise speed, altitude, exercise intensity, and the time of measurement. In multiple studies, we have shown similar reduced changes in lactate levels under acute high-altitude exposure.^6^^,^^30^ However, the mechanism underlying the lactate paradox remains debated.^31^ We revealed that resting lactate levels in the highlands were negatively correlated with cardiorespiratory fitness during CPET, which indicates that lower lactate levels are associated with better cardiorespiratory fitness during high-altitude acclimatization. Therefore, resting serum lactate levels can predict cardiorespiratory fitness during hypoxia, such as VO_2_max, VO_2_/WR slope, and VE, even in the absence of CPET equipment. This provides a convenient and relatively accurate method for assessing the cardiorespiratory fitness in a large population over a short period.

### Ubiquinol

There incidence and severity of AMS vary across studies.^32^ The development of AMS has been linked to factors such as arrival altitude, ascent speed, exercise intensity, ethnicity, age, and sex.^32^^,^^33^ In the present study, AMS incidence was high, but severity remained low, a pattern commonly observed in individuals flying to high altitudes.^32^ The lack of sufficient acclimatization to hypobaric hypoxia leads to an increased incidence of AMS. Furthermore, we propose that AMS severity may be exacerbated by greater hypoxemia and is more prevalent in individuals engaging in high-intensity activities, such as rapid hiking to high altitudes, rather than air travel.

The serum level of ubiquinol decreases with age. This leads to reduced physical performance, rapid fatigue, and weakened cellular protection against hypoxia-induced stress.^7^ Supplementation of ubiquinol markedly maintained VO_2_max and relieved fatigue during acute hypoxia.^34^ In the present study, individuals in the ubiquinol group exhibited a trend of lower AMS incidence during the first 3 days at high altitudes, although no statistical difference was observed. Since fatigue is a key criterion for AMS scoring, the fatigue symptom scores in the ubiquinol group followed a similar trend as AMS incidence. Therefore, we hypothesize that the potential benefits of ubiquinol in alleviating fatigue may account for this trend. Ubiquinol supplementation may help reduce AMS incidence by relieving fatigue.

Peak CO and SpO_2_ are determinants of oxygen transport efficiency in the circulatory system during maximal exercise. A decline in these parameters may primarily contribute to the reduction in VO_2_max at high altitudes. However, the increased VO_2_max was not accompanied by changes in Peak CO or SpO_2_ in individuals taking ubiquinol. This suggests that ubiquinol may increase VO_2_max via other mechanisms, such as promoting oxygen utilization efficiency in the mitochondria and increasing the arteriovenous oxygen difference. The following mechanisms may account for the beneficial effects of ubiquinol on high-altitude cardiorespiratory fitness. Hypoxia reduces complex I activity in cells, causing accumulation of the electron carrier ubiquinol, which drives the succinate dehydrogenase complex and enables electron deposition onto fumarate.^35^^,^^36^ Fumarate reduction sustains dihydroorotate dehydrogenase and complex I activity. Ubiquinol administration promotes electron deposition in the fumarate and electron transport chain. Ubiquinol supplementation before hypoxia could result in cellular accumulation, creating a potential electron flow in the electron transport chain that maintains mitochondrial function under hypoxic conditions. Furthermore, ubiquinol supplementation in the highlands showed significant antioxidative and anti-inflammatory effects, which might contribute to its beneficial effects during acclimatization.

### Conclusions

As shown in graphical abstract, this study elucidates the characteristics of cardiorespiratory fitness during high-altitude acclimatization and deacclimatization, indicating that short-term journeys to the highlands do not significantly affect cardiorespiratory fitness or physical performance capacity after return. It also revealed desynchronization between cardiovascular and respiratory recovery during deacclimatization. We show that oral ubiquinol supplementation before high-altitude exposure helps maintain cardiorespiratory fitness and alleviates fatigue during acclimatization. Although these findings need to be validated in a large cohort, they represent a potential pharmacological intervention for more than 100 million people who experience high-altitude exposure every year.

### Limitations of the study

Although some findings of this trial are inspiring, there are some limitations to consider. First, the capacity to adapt to high-altitude hypoxia differs among individuals of different nationalities, with Sherpas and Andean Indians exhibiting enhanced high-altitude acclimatization capacity. All our volunteers were Han Chinese; therefore, our conclusions may not be applicable to highland natives. Second, all volunteers were healthy adults (aged 18–55 years). Therefore, cardiorespiratory fitness and the effect of ubiquinol in populations with cardiopulmonary abnormalities, as well as in adolescents and older individuals, require further investigation. Large-scale studies are required to validate these findings.

## Resource availability

### Lead contact

Further information and requests for resources and reagents should be directed to and will be fulfilled by the lead contact, Jie Yang (yangjie0818@hotmail.com).

### Materials availability

This study did not generate new unique reagents.

### Data and code availability

• All data have been deposited at [Dataverse: https://doi.org/10.7910/DVN/ECVZDF] and are publicly available as of the date of publication.
• This paper does not report original code.
• Any additional information required to reanalyze the data reported in this paper is available from the lead contact upon request.

## Acknowledgments

We are forever grateful to the late Prof. Lan Huang for his pioneer work and revolutionary theories in high-altitude cardiovascular medicine.

We thank all the volunteers in this clinical trial and the Shigatse Hospital in Tibet. This work was supported by Noncommunicable Chronic Diseases-National Science and Technology Major Project (2024ZD0526900, 2024ZD0526902), Chongqing Talents: Exceptional Young Talents project (J.Y.), the Program for Excellent Talents in Army Medical University (grant number 2022XKRC008, J.Y.), and the project for Young Talented Doctors of Xinqiao Hospital Affiliated to Army Medical University (no. 2022YQB080).

## Author contributions

J.Y. and L.H. participated in the funding acquisition, project administration, and supervision. J.Y. contributed to the investigation, writing–original draft, and visualization of the abstract. H.L. contributed to the manuscript revision. Z.L., H.L., and M.S. participated in formal analysis, validation, investigation, and analysis. S.Y. and M.H. contributed to the conceptualization and methodology. S.B. and X.Y. contributed to this investigation. K.W. contributed to data curation. H.D., B.Y., and C.Z. contributed to writing—review, and editing. Most of the authors flew to 3900 m and returned to 300 m with all volunteers. All authors contributed to the revision of the manuscript and reviewed and approved the final version.

## Declaration of interests

The authors declare no competing interests.

## STAR★Methods

### Key resources table

**Table:** 

REAGENT or RESOURCE	SOURCE	IDENTIFIER
**Software and algorithms**		

G∗ Power 3.1.9.2	Germany	https://www.ncss.com/download/pass/
SPSS 26.0	IBM, Armonk, NY, USA	https://www.ibm.com/cn-zh/spss

**Other**		

Electronically braked cycle ergometer	Custo Med Gmbh, Ottobrunn, Germany	EC3000e
Spiroergometric system with breath-by-breath technology	Cortex Biophysik, Leipzig, Germany	MetaLyzer 3B
Automated hematology corpuscle analyzer	Mindray Co., Ltd., Shenzhen, China	BC-3000 plus

### Experimental model and study participant details

#### Ethics statement

This randomized, double-blind, placebo-controlled clinical trial (Shigatse CARdiorespiratory Fitness [SCARF] Trial) was conducted at Chongqing Xinqiao Hospital and Shigatse Branch Hospital in 2022. The study was approved by the Medical Ethics Committee of Xinqiao Hospital of Army Medical University (approval No. 2022-060-01) and was performed according to the recommendations of the Declaration of Helsinki. All participants provided written informed consent prior to initiating any study-related procedure.

#### Study participant details, randomization blinding, and initial visit

The sample size was calculated using G∗ Power (version 3.1.9.2, Germany). Based on a previous cardiorespiratory fitness study,^24^ the effect size, α error probability, and power (1 − β) were set to 0.25, 0.05, and 0.9, respectively. Considering the correlation among repeated-measures analysis of variance (ANOVA) of 0.5, a sample size of 36 was required to evaluate the effects of ubiquinol. Considering a 5% dropout rate, 38 participants were required across the two groups (19 in each group) and within the interactions.

The study protocol was published previously.^37^ Healthy Chinese Han volunteers, aged 18–55 years, who had resided in lowlands (<500 m) for at least 10 years without recent exposure to highlands (>2,500 m) in the last six months were included. Candidates who had recently taken nonsteroidal anti-inflammatory drugs, acetaminophen, diuretics, or any other medication for the prevention or treatment of acute mountain sickness (AMS) were excluded. A total of 41 subjects (15 males and 26 females) participated in the study.

The study employed a computer-generated block randomization sequence to allocate 41 eligible participants in a 1:1 ratio. A total of 20 patients were randomly assigned to the experimental group and 21 patients to the control group. The research coordinators provided a randomization number to the research assistants after obtaining written consent from and enrolling eligible participants. The randomization process was independently managed by a research coordinator not involved in patient recruitment or outcome assessment to ensure allocation concealment. The participants, laboratory staff, data gatherers, and statistical analysts remained blinded to group allocation throughout the study. Treatment allocation was concealed in an opaque envelope until opened on the day of treatment. Allocation was not made until the end of data analysis or until adverse events such as severe diarrhea occurred. The participants were reminded daily of the medications via email. The remaining medications were returned to the researchers to improve monitoring and compliance.

### Method details

#### Clinical visits

All participants were requested to take two capsules daily, constituting a 200 mg ubiquinol dose or placebo, for 14 consecutive days before departure to the highlands (graphical abstract and Figure S3). All tests were performed in a temperature-controlled (20–25°C) laboratory by a physician and technician, who were blinded to treatment allocation and other outcome assessments. Each participant underwent CPET, routine blood and serum examination, and completed a questionnaire at three visits: 3 days before leaving Chongqing, China (300 m altitude); 3 days after arriving in Shigatse, China (3,900 m altitude), and 7 days after returning to Chongqing (Figure S3). No harmful or unintended effects were observed in either group.

#### Respiratory function assessment and cardiopulmonary exercise testing

Standardized assessments of resting lung function and maximum effort incremental-ramp CPET were performed using an electronically braked cycle ergometer (EC3000e, Custo Med Gmbh, Ottobrunn, Germany) employing a spiroergometric system with breath-by-breath technology (MetaLyzer 3B, Cortex Biophysik, Leipzig, Germany), as previously described.^30^ The cycle ergometry exercise consisted of 3 min of rest, 3 min of unloaded exercise, a maximum incremental ramp (20 or 25 W/min), and recovery with 3 min of unloaded cycling. The main CPET indicators in this study were measured as follows: CO was estimated using the indirect Fick method employing the following equation: CO = VCO_2_/(CvCO_2_−CaCO_2_). The concentrations of CO_2_ in mixed venous (CvCO_2_) and arterial blood (CaCO_2_) were calculated based on the partial pressure in the gas phase. One MET was defined as the consumption of 3.5 mL oxygen per kilogram of body weight per minute. The VO_2_/WR slope was measured as the slope of the regression line of VO_2_ versus the work rate. The peak stage was determined when there was no further (or a relatively small) increase in VO_2_, despite further increases in the WR. Symptom limitation during an incremental exercise protocol was considered to reflect the peak stage, even though a plateau in VO_2_ failed to appear. The parameters for peak were obtained at this time point. Detailed definitions and methods for calculation of other cardiopulmonary indicators have been described previously.^37^ The standards of the European Respiratory Society^38^ and American Heart Association^39^ were used to obtain and interpret the spirometry and CPET data.

#### The Lake Louise symptom scoring system and acute mountain sickness

The presence of AMS in the highlands was assessed using the 2018 version of the self-administered Lake Louise symptom (LLS) scoring system.^40^ Briefly, headache, gastrointestinal distress, fatigue/weakness, and dizziness/vertigo were scored as 0 (none), 1 (mild), 2 (moderate), or 3 (severe). A total score of ≥3 combined with headache was used to diagnose AMS.

#### High-altitude deacclimatization syndrome score

High-altitude decacclimatization symptoms (dizziness, fatigue, sleepiness, headache, insomnia, unresponsiveness, memory loss, agitation, chest tightness, decreased appetite, increased appetite, abdominal distention, diarrhea, constipation, abdominal pain, coughing, expectoration, throat pain or discomfort, lumbago, arthralgia, or flustering) were evaluated, and the HADAS score was calculated.^41^ The physicians evaluated the symptoms of altitude deacclimatization according to the following criteria: having a slight reaction that 1) did not affect daily work (0 point); 2) affected daily work, although the symptoms improved soon after rest (score 1); 3) seriously affected daily work and symptoms improved significantly after drug treatment (score 2); and 4) seriously affected daily work and symptoms did not significantly improve after drug treatment (score 3).

#### Blood sample tests

The participants were instructed to avoid eating or drinking anything apart from water for up to 12 h. Blood samples were collected between 8:00 and 8:15 am on the day before the exercise tests at different altitudes and were divided into two parts. The first sample was analyzed using a BC-3000 plus automated hematology corpuscle analyzer (Mindray Co., Ltd., Shenzhen, China). Biochemical parameters were measured at the Clinical Laboratory of Cardiology Science at Xinqiao Hospital. Blood collected in the highlands was frozen in transfer tanks at 4°C and flown to Xinqiao Hospital within 3 h. Preservation and destruction were performed according to the hospital policies. The remaining blood samples were used to measure circulating parameters using a high-sensitivity enzyme-linked immunosorbent assay (Jiangsu Jingmei Biological Technology Co., Ltd., Jiangsu, China) in the lowlands, after ascent to the highlands, and after descent back to the lowlands.

#### Endpoints

The primary endpoints were changes in cardiorespiratory fitness, cardiovascular and respiratory system responses, and effects of ubiquinol during high-altitude acclimatization and subsequent deacclimatization. The secondary endpoints included AMS symptoms, HADAS scores, and a range of blood biochemical parameters.

### Quantification and statistical analysis

#### Statistical methods

Based on the results of the shapiro–wilk test, continuous variables were determined whether they followed a normal distribution and expressed as means ± standard deviation or median (interquartile range). Categorical variables are expressed as numbers (%). The Mann–Whitney *U*-test and independent-samples *t*-test (two-sided) were used to statistically compare the continuous variables between the groups and the results are demonstrated in all Table and Figures 1 and 2. Chi-square test was used to compare the categorical variables between the groups and the results are displayed in Table 1. As shown in Tables 3 and 4, repeated-measures ANOVA was used to account for repeated observations if the data were normally distributed; otherwise, generalized estimating equations with an independent working correlation were used. In the mixed models, treatment, altitude, and treatment × altitude interactions were considered independent variables. Treatment and altitude effects were tested if no significant interaction was observed. Otherwise, simple effects tests were used to evaluate the between-group differences at each altitude. Least significant difference tests were performed to adjust for within-altitude differences in multiple comparisons. Cardiorespiratory fitness-associated parameters were corrected for sex. Correlation analyses between continuous variables were performed using Pearson’s correlation coefficient, whereas Spearman’s rank was used for non-normally distributed variables and the results are demonstrated in Figure 3. Two-sided tests with *p*-values <0.05 were considered statistically significant. Statistical analyses were performed using the SPSS statistics software for windows (version 26, IBM, Armonk, NY, USA).

### Additional resources

This research is registered at www.chictr.org.cn (ChiCTR2200059900).
