# Supplementary material for: Cardiorespiratory fitness and effects of ubiquinol during high-altitude acclimatization and deacclimatization: The SCARF trial
Source: iScience. 2025 Feb 27;28(3):112112. doi: 10.1016/j.isci.2025.112112 (PMC11952777; doi:10.1016/j.isci.2025.112112)
Supplement: Document S1. Figures S1–S3 and Tables S1–S3 [file mmc1.pdf]

iScience, Volume *28*

Supplemental information

Cardiorespiratory fitness and effects of ubiquinol during high-altitude acclimatization and deacclimatization: The SCARF trial

Hailin Lv, Zhen Liu, Mengjia Sun, Shiyong Yu, Mingdong Hu, Shizhu Bian, Xiaowei Ye, Ke Wang, Hongmei Dong, Bingjie Yang, Chao Zhou, Lan Huang, and Jie Yang

Supplementary Figures

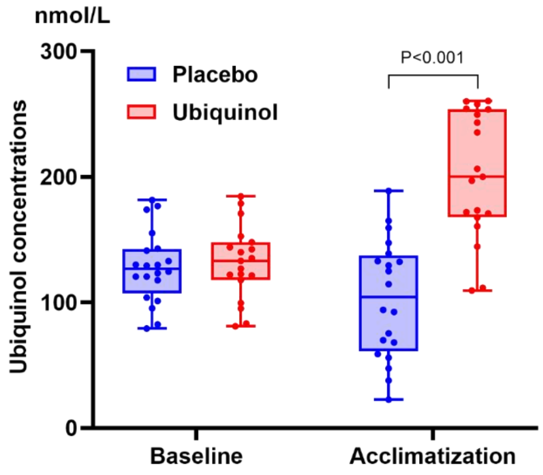

Figure S1. Serum concentration of ubiquinol in the two groups on day 0 (before treatment) and day 17 (after treatment).

Data are represented as means ± standard deviation. The independent-samples t-test (two-sided) were used to statistically compare the continuous variables. Oral supplementation of ubiquinol significantly increased the serum concentration of ubiquinol in the ubiquinol group compared with that in the placebo group (201.43 ± 49.21 vs. 102.79 ± 45.77 nmol/L, $P < 0.001$).

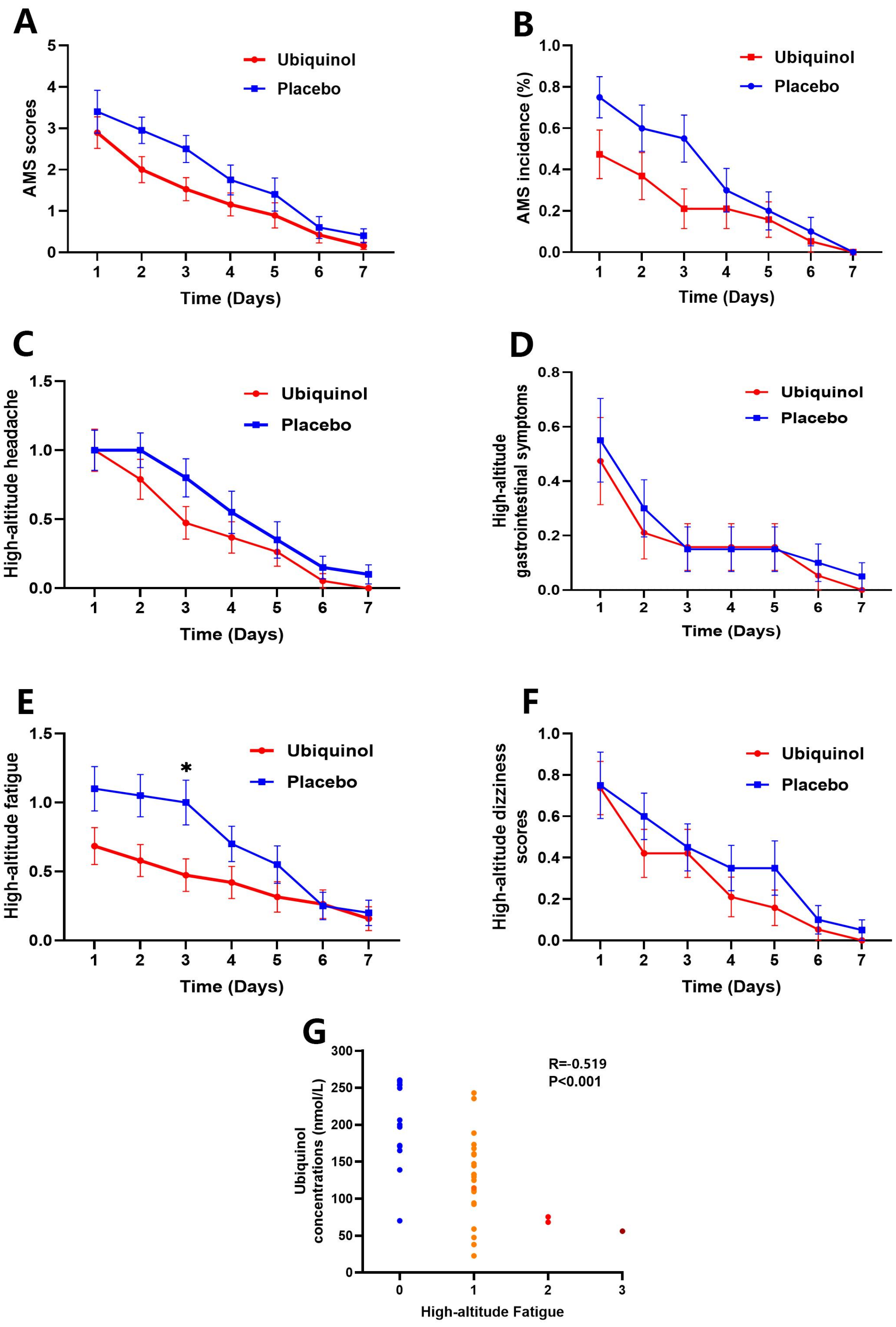
A
AMS scores
Ubiquinol
Placebo
Time (Days)
B
AMS incidence (%)
Ubiquinol
Placebo
Time (Days)
C
High-altitude headache
Ubiquinol
Placebo
Time (Days)
D
High-altitude gastrointestinal symptoms
Ubiquinol
Placebo
Time (Days)
E
High-altitude fatigue
*
Ubiquinol
Placebo
Time (Days)
F
High-altitude dizziness scores
Ubiquinol
Placebo
Time (Days)
G
Ubiquinol concentrations (nmol/L)
R=-0.519
P<0.001
High-altitude Fatigue

Figure S2. AMS and related symptoms in the ubiquinol and placebo groups during high altitude (n = 39).

(A–F) The AMS scores (A), AMS incidence (B), and scores of headache (C), gastrointestinal symptoms (D), fatigue (E) and dizziness (F) were compared in the placebo and ubiquinol groups during the first week of high-altitude exposure.

(G) The fatigue scores were negatively correlated with the ubiquinol concentrations. Data are represented as means ± standard deviation. The Mann-Whitney U-test and independent-samples t-test (two-sided) were used to statistically compare the continuous variables. *$P < 0.05$ was considered statistically significant. AMS: acute mountain sickness.

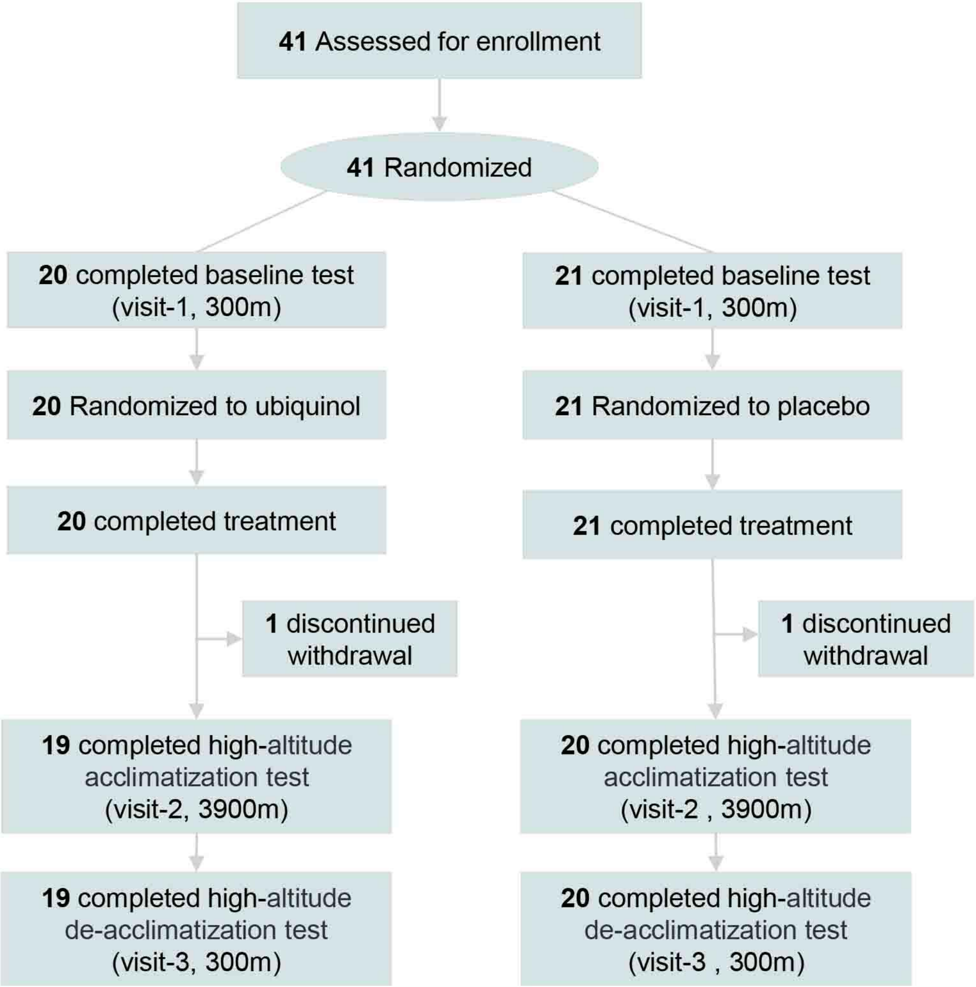

Figure S3. Flow diagram.

The participants were instructed to take ubiquinol or placebo orally 14 days before departure to a high-altitude region. All randomized participants who completed the CPET, blood examination, and questionnaires over the subsequent three visits were included in the efficacy analyses. Two participants (ubiquinol, n = 1; placebo, n = 1) who discontinued did not undergo CPET during high-altitude acclimatization. CPET: cardiopulmonary exercise testing.

S1 Table. Sex effects during high-altitude acclimatization and de-acclimatization.

Variables	Baseline			Acclimatization			De-acclimatization			P1	P2	P3	interaction effect
	Male (n = 14)	Female (n = 25)	P	Male (n = 14)	Female (n = 25)	P	Male (n = 14)	Female (n = 25)	P				
Peak VO_2 (mL/min/kg)	33.4±5.3	28.9±4.3	0.006	28.9±5.8	24.1±5.4	0.013	33.1±6.3	28.0±4.8	0.007	<0.001	0.152	<0.001	0.860
Peak METS	9.5±1.6	9.2±1.7	0.448	8.3±1.7	7.6±1.7	0.272	9.4±1.8	8.8±1.5	0.276	<0.001	0.081	<0.001	0.724
Peak RER	1.20±0.07	1.21±0.08	0.592	1.05±0.05	1.14±0.07	0.483	1.23±0.09	1.19±0.07	0.114	<0.001	<0.001	0.955	0.183
RER at AT	0.84 (0.81–0.88)	0.84 (0.80–0.86)	0.355	0.90 (0.87–0.96)	0.88 (0.86–0.91)	0.318	0.86 (0.84–0.89)	0.83 (0.79–0.87)	0.168	<0.001	0.290	<0.001	0.908
VO_2 at AT (mL/min/kg)	15..0 (11.8–17.0)	16.0 (13.5–17.5)	0.442	15.0 (13.0–17.0)	14.0 (12.0–16.0)	0.461	16.0 (14.8–17.3)	14.0 (13.0–17.5)	0.323	0.024	0.963	0.022	0.726
VO_2 at RCP (mL/min/kg)	28.3±5.7	26.8±4.0	0.362	25.1±5.7	21.3±4.8	0.030	28.1±5.6	25.0±4.6	0.075	<0.001	0.031	<0.001	0.147
VO_2/ WR slope (mL/min/W)	9.7±0.8	9.1±1.0	0.066	9.1±0.9	7.7±1.1	<0.001	9.3±1.0	9.1±1.1	0.438	<0.001	0.177	<0.001	0.005
SpO_2 at rest (%)	96.0 (95.0–97.0)	97.0 (96.0–98.0)	0.177	85.5 (83.8–89.0)	81.0 (79.5–84.0)	0.002	97.0 (96.0–97.3)	97.0 (96.0–97.5)	0.487	<0.001	0.462	<0.001	<0.001
SpO_2 at AT (%)	96.0 (95.0–97.0)	97.0 (95.0-97.5)	0.708	83.5 (79.0-85.3)	81.0 (76.5-83.0)	0.122	96.0 (95.0-97.0)	97.0 (96.5-98.0)	0.010	<0.001	0.147	<0.001	0.017
SpO_2 at RCP (%)	96.0 (94.0–97.5)	97.0 (95.8–98.0)	0.165	80.0 (77.5–83.0)	81.5 (77.8–84.3)	0.724	97.0 (94.5–97.5)	96.0 (94.0–97.3)	0.465	<0.001	0.899	<0.001	0.410
Peak SpO_2 (%)	96.0 (95.0–96.3)	95 (91.5–96.0)	0.275	81.0 (77.5–85.3)	80.0 (78.5–84.0)	0.659	95.0 (91.3–97.3)	96.0 (91.0–97.0)	0.988	<0.001	0.716	<0.001	0.388

Values are given as the mean ± standard deviation or median (interquartile range).

P1, The difference between baseline and acclimatization; P2, The difference between baseline and de-acclimatization; P3, The difference between acclimatization and de-acclimatization. VO_2, oxygen uptake; METs, metabolic equivalents; RER, respiratory exchange ratio; AT, anaerobic threshold; RCP, respiratory compensation point; WR, work rate, SpO2, Peripheral blood oxygen saturation.

S2 Table. Cardiovascular responses to high-altitude acclimatization and de-acclimatization.

Variables	Baseline			Acclimatization			De–acclimatization			P1	P2	P3	interaction effect
	Placebo (n = 20)	Ubiquinol (n = 19)	P	Placebo (n = 20)	Ubiquinol (n = 19)	P	Placebo (n = 20)	Ubiquinol (n = 19)	P				
HR at rest (bpm), mean (SD)	80.6±7.7	77.0±11.1	.238	96.2±9.7	92.8±11.3	.325	80.5±7.4	76.6±8.0	.130	<.001	.870	<.001	.991
SBP at rest (mmHg), median (IQR)	112 (103–121)	106 (103–121)	.694	120 (101–127)	118 (108–123)	.868	114 (105–121)	111 (102–116)	.101	<.001	.506	.002	.507
DBP at rest (mmHg), mean (SD)	73.6±11.8	74.6±8.6	.734	77.6±13.4	79.8±10.7	.772	73.3±14.0	70.6±9.7	.462	.014	.233	.001	.427
CO at rest (L/min), median (IQR)	3.7 (3.4–4.7)	3.6 (3.1–4.6)	.368	3.4 (3.0–4.7)	3.8 (3.3–4.9)	.390	3.8 (3.3–4.7)	3.5 (3.2–4.6)	.880	.634	.783	.475	.016
O_2 pulse at AT (mL), median (IQR)	7.5 (6.3–8.8)	8.0 (7.0–9.0)	.721	6.0 (5.0–8.8)	6.0 (5.0–7.0)	.655	7.5 (7.0–10.0)	8.0 (6.0–9.0)	.858	<.001	.415	<.001	.905
Peak HR (bpm)	166.2±12.5	167.1±11.3	.824	156.3±15.9	168.2±12.3	.013	163.8±15.3	166.4±13.8	.570	.060	.420	.274	.084
Peak O_2 pulse (mL), median (IQR)	10.5 (9.0–14.8)	10.0 (8.0–12.0)	.924	8.0 (7.0–12.0)	9.0 (8.0–12.0)	.238	10.5 (9.0–12.0)	10.0 (8.0–12.0)	.545	<.001	.167	<.001	.491
Peak CO (L/min), median (IQR)	10.1 (8.7–13.6)	10.0 (8.8–14.0)	.992	8.8 (6.4–11.6)	8.4 (7.6–13.0)	.329	9.9 (8.6–12.3)	9.4 (8.3–13.1)	.798	<.001	.180	<.001	.036
Peak CP (mmHg/L/min), median (IQR)	4.6 (4.0–5.2)	4.7 (3.8–5.4)	.663	3.5 (2.8–4.0)	4.1 (3.5–4.6)	.077	4.5 (3.8–5.3)	3.9 (3.3–5.0)	.544	<.001	.107	<.001	<.001
HR recovery of 1 min (bpm), median (IQR)	23 (16–28)	27 (20–30)	.161	18 (15–22)	17 (12–24)	.730	23 (18–32)	27 (22–37)	.108	<.001	.091	<.001	.518
HR recovery of 2 min (bpm), median (IQR)	21 (17–27)	22 (19–25)	.153	18 (14–21)	15 (14–21)	.633	19 (17–24)	22 (17–24)	.126	<.001	.936	<.001	.058

Values are given as the mean ± SD or median (IQR).

P1: difference between baseline and acclimatization; P2: difference between baseline and de-acclimatization; P3: difference between acclimatization and de-acclimatization; HR: heart rate; SBP: systolic blood pressure; DBP: diastolic blood pressure; CO: cardiac output; CP: circulatory power; AT: anaerobic threshold; SD: standard deviation; IQR: interquartile range.

S3 Table. Respiratory responses during high-altitude acclimatization and de-acclimatization.

Variables	Baseline			Acclimatization			De–acclimatization			P1	P2	P3	Interaction effect
	Placebo (n = 20)	Ubiquinol (n = 19)	P	Placebo (n = 20)	Ubiquinol (n = 19)	P	Placebo (n = 20)	Ubiquinol (n = 19)	P				
FEV_1/FVC (%), median (IQR)	75 (60–85)	78 (68–86)	.495	78 (69–86)	81 (72–83)	.942	80 (70–88)	84 (68–92)	.480	.026	.006	.453	.707
VE at rest (L), median (IQR)	10.3 (7.9–12.0)	8.5 (7.4–10.6)	.142	11.8 (9.1–13.7)	10.7 (9.0–13.6)	.801	10.1 (9.1–12.7)	9.5 (8.1–11.6)	.335	<.001	.068	<.001	.264
MVV (L/min), mean (SD)	126.1±32.6	129.3±34.1	.768	136.7±30.3	148.8±41.4	.304	122.9±33.6	126.8±35.6	.729	<.001	.472	<.001	.385
VE/MVV at rest (%), median (IQR)	8.5 (7.0–10.0)	8.0 (7.0–9.0)	.410	10.0 (8.3–12.0)	10.0 (9.0–12.0)	.975	9.0 (9.0–11.0)	8.0 (8.0–10.0)	.248	<.001	.225	<.001	.594
PEF (L), mean (SD)	6.3±2.0	7.1±2.5	.227	7.7±2.1	8.5±2.6	.350	7.5±2.6	8.3±2.9	.347	<.001	.001	.505	.963
FEF 25% (L/s), mean (SD)	5.5±1.8	6.0±2.3	.451	6.8±1.8	7.3±2.3	.449	6.2±2.0	7.2±2.3	.146	<.001	.002	.167	.472
FEF 50% (L/s), mean (SD)	3.9±1.4	4.0±1.2	.758	4.7±1.3	4.5±1.5	.660	4.0±1.2	4.7±1.2	.090	.001	.053	.124	.068
$PetCO_2$ at rest (mmHg), median (IQR)	30 (29–33)	32 (30–33)	.900	26 (23–27)	25 (24–27)	.598	29 (27–32)	31 (27–33)	.510	<.001	<.001	<.001	.866
Peak VE (L), median (IQR)	63 (51–78)	64 (51–83)	.903	61 (52–85)	69 (64–102)	.096	61 (52–82)	68 (52–92)	.383	<.001	.105	.039	.006
VE/VCO_2 slope, mean (SD)	27.0±2.9	28.5±4.0	.189	40.7±5.0	40.0±5.4	.667	29.3±3.6	31.7±4.6	.084	<.001	<.001	<.001	.111
Peak VE/VO_2, mean (SD)	34.1±3.5	34.3±5.2	.882	43.0±4.9	46.5±6.1	.051	35.3±5.3	37.5±5.6	.214	<.001	.014	<.001	.164
Peak VE/VCO_2, mean (SD)	29.2±2.7	30.1±3.5	.385	39.8±4.1	41.2±4.9	.304	32.2±3.9	33.6±4.3	.291	<.001	<.001	<.001	.883

Peak VD/VT (%), mean (SD)	9.0±1.7	9.0±3.0	.661	6.9±3.1	7.0±3.0	.830	10.0±1.4	10.0±3.0	.535	<.001	.003	<.001	.925
Peak $PetCO_2$ (mmHg), median (IQR)	37 (34–40)	35 (32–40)	.497	26 (24–28)	25 (24–26)	.566	34 (32–35)	33 (30–36)	.550	<.001	<.001	<.001	.953
$PetCO_2$ at AT (mmHg), mean (SD)	38.0±3.1	37.7±2.5	.726	27.4±2.4	28.0±2.8	.482	35.5±2.4	35.0±3.1	.579	<.001	<.001	<.001	.517
VE/VO_2 at AT, median (IQR)	23.7 (21.7–24.8)	23.3 (20.7–24.3)	.338	32.7 (29.7–34.9)	31.7 (28.1–32.5)	.061	24.7 (23.4–26.7)	25.4 (23.3–26.9)	.949	<.001	<.001	<.001	.154
VE/VCO_2 at AT, median (IQR)	27.8 (26.5–29.5)	27.2 (25.8–29.8)	.603	36.2 (34.8–37.2)	35.4 (33.4–37.2)	.123	29.8 (28.2–30.7)	29.9 (28.1–31.7)	.677	<.001	<.001	<.001	.242
Peak VE/MVV (%), median (IQR)	55 (47–62)	56 (50–67)	.272	60 (47–66)	76 (67–81)	.026	53 (45–72)	64 (55–72)	.221	.001	.071	.034	.316

Values are given as the mean ± SD or median (IQR).

P1: difference between baseline and acclimatization; P2: difference between baseline and de-acclimatization; P3: difference between acclimatization and de-acclimatization. FEV_1: forced expiratory volume in 1 second; FVC: forced vital capacity; VE: minute ventilation; MVV: maximum voluntary ventilation; PEF: peak expiratory flow; FEF: forced expiratory flow; $PetCO_2$: end-tidal partial pressure of carbon dioxide; VO_2: oxygen uptake; VCO_2: carbon dioxide output; VD/VT: physiological dead space/tidal volume; AT: anaerobic threshold; SD: standard deviation; IQR: interquartile rang
